# Antifouling Marine Coatings with a Potentially Safer and Sustainable Synthetic Polyphenolic Derivative

**DOI:** 10.3390/md20080507

**Published:** 2022-08-05

**Authors:** Ana R. Neves, Luciana C. Gomes, Sara I. Faria, João Sousa, Raquel Ruivo, Inês Páscoa, Madalena Pinto, Emília Sousa, Miguel M. Santos, Elisabete R. Silva, Marta Correia-da-Silva, Filipe Mergulhão

**Affiliations:** 1 Laboratory of Organic and Pharmaceutical Chemistry, Faculty of Pharmacy, University of Porto, Rua Jorge de Viterbo Ferreira 228, 4050-313 Porto, Portugal; 2 Interdisciplinary Centre of Marine and Environmental Research (CIIMAR), University of Porto, Novo Edifício do Terminal de Cruzeiros do Porto de Leixões, Av. General Norton de Matos, s/n, 4450-208 Matosinhos, Portugal; 3 LEPABE—Laboratory for Process Engineering, Environment, Biotechnology and Energy, Faculty of Engineering, University of Porto, Rua Dr. Roberto Frias, 4200-465 Porto, Portugal; 4 ALiCE—Associate Laboratory in Chemical Engineering, Faculty of Engineering, University of Porto, Rua Dr. Roberto Frias, 4200-465 Porto, Portugal; 5 Department of Biology, FCUP—Faculty of Sciences, University of Porto, 4169-007 Porto, Portugal; 6 BioISI—Biosystems & Integrative Sciences Institute, Faculty of Sciences, University of Lisboa, Campo Grande, 1749-016 Lisboa, Portugal; 7 Departamento de Química e Bioquímica, Faculdade de Ciências, Universidade de Lisboa, Campo Grande, 1749-016 Lisboa, Portugal

**Keywords:** gallic acid, anti-biofilm, marine bacteria, endocrine disruptor assessment, safer chemicals

## Abstract

The development of harmless substances to replace biocide-based coatings used to prevent or manage marine biofouling and its unwanted consequences is urgent. The formation of biofilms on submerged marine surfaces is one of the first steps in the marine biofouling process, which facilitates the further settlement of macrofoulers. Anti-biofilm properties of a synthetic polyphenolic compound, with previously described anti-settlement activity against macrofoulers, were explored in this work. In solution this new compound was able to prevent biofilm formation and reduce a pre-formed biofilm produced by the marine bacterium, *Pseudoalteromonas tunicata*. Then, this compound was applied to a marine coating and the formation of *P. tunicata* biofilms was assessed under hydrodynamic conditions to mimic the marine environment. For this purpose, polyurethane (PU)-based coating formulations containing 1 and 2 wt.% of the compound were prepared based on a prior developed methodology. The most effective formulation in reducing the biofilm cell number, biovolume, and thickness was the PU-based coating containing an aziridine-based crosslinker and 2 wt.% of the compound. To assess the marine ecotoxicity impact of this compound, its potential to disrupt endocrine processes was evaluated through the modulation of two nuclear receptors (NRs), peroxisome proliferator-activated receptor γ (PPARγ), and pregnane X receptor (PXR). Transcriptional activation of the selected NRs upon exposure to the polyphenolic compound (10 µM) was not observed, thus highlighting the eco-friendliness towards the addressed NRs of this new dual-acting anti-macro- and anti-microfouling agent towards the addressed NRs.

## 1. Introduction

The development of innovative and eco-friendly technologies to combat marine biofouling is critical given the associated economic, environmental, and human health consequences [1,2]. Although the addition of biocides to marine coatings has been the most used and effective solution to avoid marine biofouling, the biocides currently used for this purpose are persistent, bioaccumulative, and can be toxic to some non-target marine organisms [3,4].

**Figure 1:**
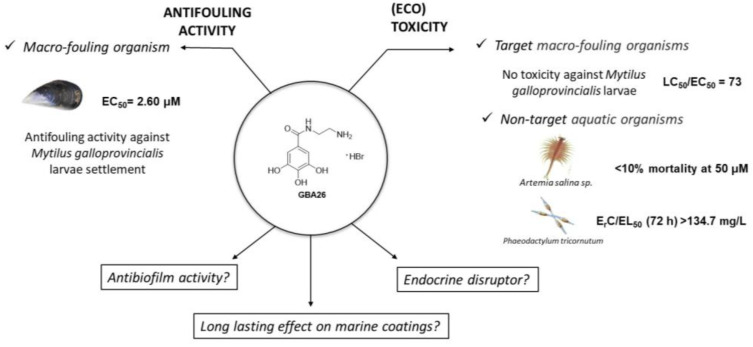
Aims of this study and antifouling activity and (eco)toxicity previously described for GBA26 [12].

When a surface is submerged in water, the marine biofouling process is triggered by the accumulation and physical adsorption of organic molecules. This conditioning film creates the perfect environment for the settlement and growth of pioneer bacteria, which leads to the formation of a biofilm matrix. This initial process leads to the so-called secondary colonization, where a biofilm of multicellular species is formed. Tertiary colonization occurs with the capture of particles and organisms, such as the larvae of marine macro-organisms, including macroalgae, sponges, cnidarians, polychaetes, mollusks, barnacles, bryozoans, and tunicates [5]. In the last five years, several antifouling (AF) compounds capable of inhibiting the settlement of the macrofouler, *Mytilus galloprovincialis* (mussel) larvae, were synthesized by some of us [6,7,8,9,10,11]. After structure-AF activity relationship studies on several gallic acid derivatives, we recently discovered that *N*-(2-aminoethyl)-3,4,5-trihydroxybenzamide hydrobromide (GBA26) (Figure 1) exhibited an optimized potency against the larvae settlement of this mussel with higher LC_50_/EC_50_ than the previous parent compound and the emerging biocide Econea^®^ (tralopyril) [12]. This property was also maintained after the incorporation of GBA26 (1 wt.%) in a polyurethane (PU)-based marine coating [12]. The IUCN/SSC Invasive Species Specialist Group has listed *M. galloprovincialis* among the 100 “World’s Worst” invaders, highlighting the relevance of this species around the world. On the other hand, mussels can be considered a target and non-target organism [13,14], making these results even more relevant. On the coastal shores, mussels represent a key species operating as ecosystem engineers by providing habitats for other organisms, filtering out sediments and pollutants, and providing food for higher trophic levels.

However, as bioactivity against a single hard fouler may not be seen in assays against soft fouling, in this work, the anti-biofilm performance of GBA26 in a solution was studied to assess if this compound is also capable of preventing the formation and/or reduction of biofilms of a common marine microfouler, *Pseudoalteromonas tunicata* (Figure 1). This organism was applied for the assessment of the in vitro AF performance of surfaces and coatings [15,16,17,18]. Hence, new PU-based coating formulations containing GBA26 aziridine-based crosslinker (CL) to enable higher service life of coatings [19] were evaluated for *P. tunicata* biofilm growth (Figure 1), at defined hydrodynamic settings [20] mimicking a real marine scenario.

Regarding ecotoxicity, GBA26 was previously shown to have lower toxicity than tralopyril against *Artemia salina*, a marine shrimp. While tralopyril cause 100% mortality to *A. salina*, GBA26 did not cause more than 10% mortality at the same concentration (50 µM) (Figure 1). To pursue a systematic ecotoxicological evaluation, the OECD 201 test was chosen to determine the impact of GBA26 on the growth of *Phaeodactylum tricornutum*, a marine diatom among the most common type of phytoplankton. GBA26 exhibited a safer profile than tralopyril and the previous analog (Figure 1) [12]. However, ecotoxicity could also be expressed by the ability of a compound to disrupt endocrine processes. For example, tributyltin, a booster biocide used in AF paints which was banned since 2008, is widely known to have an endocrine-disrupting action in mollusks [21,22,23]. Previous studies demonstrated that TBT, at a low ng/L range, modulates the nuclear receptor (NR) retinoid X receptor (RXR), inducing *imposex* development in gastropods [22]. SeaNine211^®^ was also found to have endocrine disrupting and reproductive impairing effects [24]. Early this year the effects of short-term exposure to tralopyril (Econea^®^) on physiological indexes and endocrine function in turbot (*Scophthalmus maximus*) were reported [25]. This negative effect should be foreseen during the development of new effective eco-friendly AF agents (Figure 1). Since NRs are ligand-activated transcription factors, participating in the regulation of numerous biological processes such as development, metabolism, and reproduction [26] we, therefore, investigated the ability of GBA26 to modulate the activity of selected NRs. Therefore, we also investigated the ability of GBA26 to modulate the activity of selected NRs.

## 2. Results and Discussion

### 2.1. Anti-Biofilm Performance of GBA26

**Figure 2:**
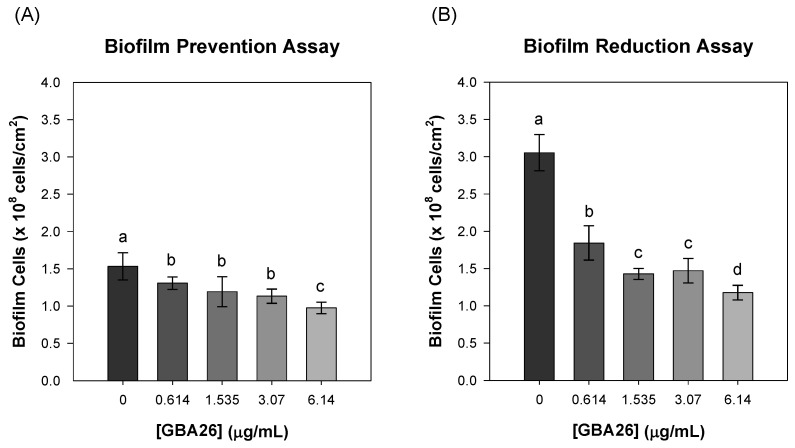
(**A**) Biofilm prevention and (**B**) biofilm reduction assays with different concentrations of GBA26. Different letters were given, when statistically significant differences existed at *p* < 0.05 (confidence level ≥ 95%). The average ± SDs for three independent assays are shown.

The in vitro anti-biofilm efficacy of GBA26, in several concentrations, was determined through a biofilm prevention assay (GBA26 mixed with inoculum) and a biofilm reduction assay (pre-formed biofilms exposed to GBA26 in solution) using *P*. *tunicata* (Figure 2).

Results of the two assays showed that GBA26 can prevent and reduce preformed biofilms of *P*. *tunicata* in a concentration-dependent manner.

### 2.2. PU-Based Coatings Containing GBA26

To assess the in vitro performance of GBA26 as an anti-biofilm agent in surface coatings, this compound was incorporated at different concentrations, approximately 1.0 to 2.0 wt.%, (Table 1) into a representative, commercial, two-component, PU-based marine coating.

Previous studies on the incorporation of GBA26 in a PU-based coating system showed that, although GBA26 had maintained AF activity against the settlement of *M. galloprovincialis* larvae, it was rapidly released from the PU-based marine coating. Therefore, to increase the service life of these coatings, further coating optimization was performed, according to an existing immobilization methodology [19], which involved the addition of the trimethylolpropane triaziridine propionate (TZA) crosslinker (CL) into the formulation. This aziridine-based crosslinker, widely used in polymeric formulations such as paints, reacts rapidly with nucleophilic functional groups of the compounds (e.g., amines, alcohols), promoting cross-links between different functional additives, and improving their compatibility with the polymeric systems [19].

The generated GBA26-based coating systems were further assessed in terms of anti-biofilm performance.

### 2.3. Pseudoalteromonas Tunicata Biofilm Formation under Defined Hydrodynamic Conditions

**Figure 3:**
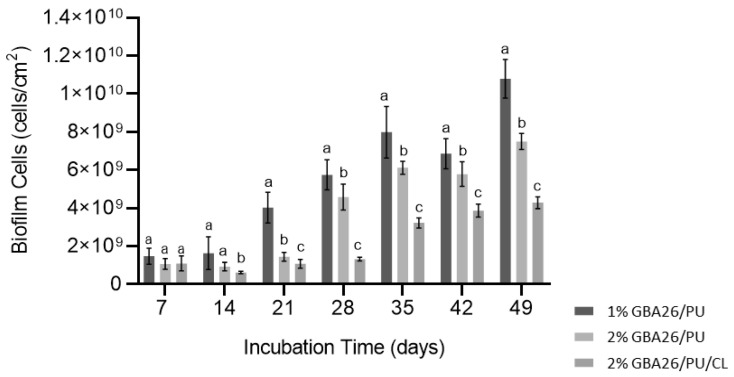
Effect of PU-based coatings containing different concentrations of GBA26 and a crosslinker (CL) on biofilm development in *Pseudoalteromonas tunicata* for 49 days. The analyzed parameter refers to biofilm cell numbers. Different letters were given, when statistically significant differences existed at *p* < 0.05 (confidence level ≥ 95%). The average ± SDs for three independent assays are shown.

Figure 3 shows the biofilm content in terms of the number of biofilm cells (cells/cm^2^) of *P. tunicata* for the three investigated coating formulations.

For the 1 wt.% GBA26 PU-based coating, the number of cells increased only from day 14 until day 49. No significant difference was observed between day 7 and day 14, suggesting that a strong anti-biofilm activity was exerted in the first 14 days.

The anti-biofilm effect was observed for the 2 wt.% GBA26 PU-based coating in the first 21 days, after which the number of biofilm cells started to increase, although never reaching the number of cells observed for the 1 wt.% GBA26 PU-based coating.

A long-lasting effect was observed for the 2 wt.% GBA26 PU-based coating containing the crosslinker (CL), GBA26/PU/CL, where the number of cells only started to increase from day 28 and the number of biofilm cells was lower (around 4 × 10^9^ cells/cm^2^) than the number of cells observed for the 2 wt.% GBA26 PU-based coating without CL at day 49 (around 8 × 10^9^ cells/cm^2^).

**Figure 4:**
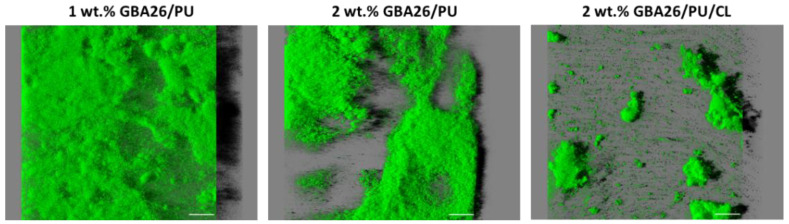
*Pseudoalteromonas tunicata* biofilm architecture on a surface protected with 1 wt.% GBA26/PU coating, a surface treated with 2 wt.% GBA26/PU coating, and a surface treated with 2 wt.% GBA26/PU/CL coating after 49 days. These images were obtained from a confocal image series and are representative of the biofilm top view, with the virtual shadow projection on the right. The white scale bar corresponds to 50 µm.

The structural differences of *P. tunicata* biofilms, which were developed on the three tested surfaces after a 49-day assay, were assessed through a confocal laser scanning microscope (CLSM); this is similar to what was recently performed by other authors for novel anti-biofilm materials for marine applications [27]. Three-dimensional (3D) images of visualized stacks are presented in Figure 4 (showing the aerial view of biofilm and including a virtual shadow projection on the right-hand side which represents the biofilm section) and Figure S1 in the Supplementary Material (isosurface rendered 3D visualizations of the same data set used to obtain Figure 4).

**Figure 5:**
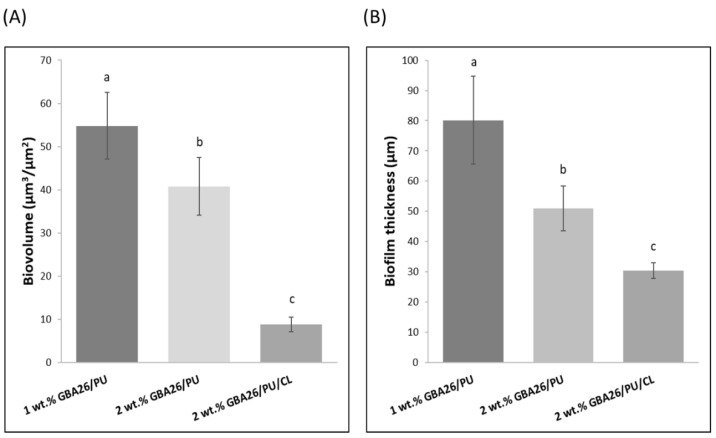
(**A**) Biofilm biovolume and (**B**) thickness of the 49-days-old biofilms extracted from the confocal z-stacks with the COMSTAT tool. Different letters were given, when statistically significant differences existed at *p* < 0.05 (confidence level ≥ 95%). The average ± SDs for three independent assays are shown.

A thicker and denser biofilm, developed on the GBA26 PU-based coating (1 wt.% GBA26/PU), confirms the results obtained from the biofilm cell count (Figure 3). On the other hand, biofilms formed on the top of the GBA26/PU/CL (2 wt.% GBA26) did not cover the entire surface area and only scattered cell aggregates could be observed. Regarding biofilm biovolumes, it was significantly lower for the GBA26/PU/CL surface when compared to 1 and 2 wt.% GBA26 PU-based coatings (*p* < 0.05, Figure 5A). Accordingly, the biofilm thickness was higher for the 1 wt.% GBA26 PU-based coating formulation than for the 2 wt.% GBA26 PU-based coating and the 2 wt.% GBA26 PU-based coating containing the CL, GBA26/PU/CL (*p* < 0.05, Figure 5B).

### 2.4. In Vitro Transcriptional Activation of HsPPARγ, DrPPARγ, and DrPXR

Given that a high number of NRs exists across the metazoans (i.e., 48 NRs in humans, 73 NRs in teleosts [28]), it is not feasible to test all NR/test compound combinations, to assess possible NR-dependent disruption mechanisms upon exposure to novel contaminants. Since GBA26 is a polyphenolic compound, and polyphenols are described in the literature as activators of both PPARγ [29] and PXR receptors, the two receptors were selected for the in vitro transactivation assays [30]. PPARγ is a master regulator of an adipogenesis, and a known target of environmental chemicals, such as the AF biocide TBT and PXR, a modulator of detoxification responses exhibiting a highly plastic ligand-binding pocket [31,32,33,34,35]. Therefore, for the present study, we addressed the ability of GBA26 to modulate PPARγ from *H. sapiens* and the teleost, *Danio rerio* and *D. rerio* PXR. 

**Figure 6:**
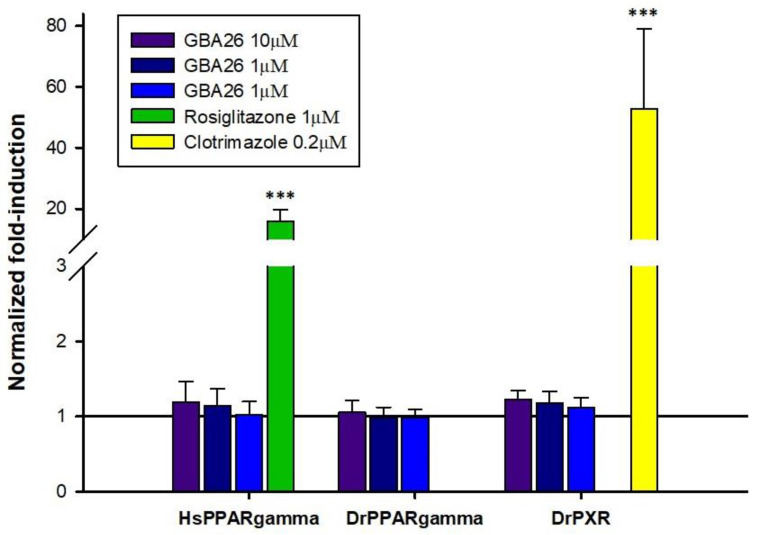
In vitro transcriptional activation of HsPPARγ, DrPPARγ, and DrPXR in the presence of 10 to 0.1 μM GBA26. Results are expressed as average fold induction relative to the vehicle control, DMSO, solid line (mean ± standard deviation). Asterisks denote significant differences (*** *p* < 0.001).

As expected, the control compounds, rosiglitazone and clotrimazole, yielded significant fold-induction values with *H. sapiens* PPARγ and *D. rerio* PXR, respectively, when compared to DMSO, confirming the validity of the present assay (Figure 6).

Overall, the results suggest that GBA26 does not modulate the transcription of the selected NRs, at least at low concentrations, suggesting that this new AF compound may not interfere with endocrine processes mediated by PPARγ (a known target of endocrine disruptors) and PXR (a highly promiscuous receptor involved in drug metabolism) in the studied species. 

## 3. Materials and Methods

### 3.1. Reagents

*N*-(2-aminoethyl)-3,4,5-trihydroxybenzamide hydrobromide (GBA26) was synthesized according to our previously described procedure [12]. *N*-methylpyrrolidone 99.5% (368450010) was purchased from Acros Organics (Geel, Belgium).

### 3.2. Anti-Biofilm Assays

Two different types of experiments were performed to determine the in vitro anti-biofilm activity of GBA26 prevention and reduction assays. For the biofilm prevention assay, a cell suspension of *P. tunicata*, at an initial concentration of 1 × 10^8^ cells/mL in the marine medium Våatanen Nine Salt Solution (VNSS), was placed in contact with different concentrations of GBA26 (0, 0.614, 1.535, 3.07, and 6.14 µg/mL) for 24 h in a 12-well polystyrene plate (VWR International, Carnaxide, Portugal). The microplate was incubated at 25 °C and 185 rpm in a 25mm diameter shaker (Agitorb 200ICP, Norconcessus, Ermesinde, Portugal) to allow the formation of biofilm in shear conditions similar to those found on a ship moored in a harbor [18]. For the biofilm reduction assay, 7-day biofilms of *P. tunicata* were first formed in VNSS in 12-well plates under the previously mentioned orbital shaking conditions and then exposed to the same concentrations of the test compound for 24 h, maintaining the hydrodynamic conditions. At the end of each assay, biofilm cells were removed from the surface and suspended in a sterile saline solution for cell counting under a light microscope (Nikon Eclipse LV100 microscope, Nikon Corporation, Tokyo, Japan). Three independent biofilm assays, with three technical replicates each, were performed.

### 3.3. Immobilization of GBA26 in a Polyurethane-Based Marine Coating

To evaluate the performance of GBA26 as an anti-biofilm agent in surface coatings, the compound was incorporated at different concentrations, close to 1.0 and 2.0 wt.%, (Table 1) into a representative, commercial, two-component, PU-based marine paint, including the base resin F0032 and the curing agents 95,580 (Hempel, A/S Copenhagen, Denmark).

For the conventional preparation of PU-based formulations, GBA26 dissolved in *N*-methyl pyrrolidone with a GBA26/solvent weight ratio of 0.38, was added and blended into the PU-based paint components in the exact amounts to yield a GBA26 content of 1.05 ± 0.01 and 1.98 ± 0.01 wt.% in the wet and uncured coating formulations, respectively (c.f. Table 1).

For the PU-based formulation containing 1.88 ± 0.01 wt.% GBA26 and the trimethylolpropane triaziridine propionate (TZA, 99.5%, PZ Global, Barcelona, Spain), the same preparation methodology was performed, but with a GBA26/solvent ratio of 0.35, and a crosslinker content of 1.08 ± 0.48 wt.% in the wet formulation.

For all prepared formulations the base and curing agent weight proportion was 9/1, according to the supplier’s instructions.

The generated GBA26-based formulations were further used to coat 1 × 1 cm^2^ glass substrates (Vidraria Lousada, Lda, Lousada, Portugal) through a conventional dipping coating procedure.

### 3.4. Dynamic Biofilm Assay

The ability of *P. tunicata* to colonize the PU-based coatings was followed for 7 weeks using 12-well microplates under the hydrodynamic conditions referred to for the anti-biofilm assays. Biofilm development was followed for 49 days because this period represents approximately half of the interval considered economically viable for the preservation of underwater systems and hull cleaning. This methodology was previously shown to provide a similar pattern of results when compared to surface immersion in real marine settings (i.e., immersion for extended periods in the Atlantic) [36]. A *P. tunicata* suspension of 1 × 10^8^ cells/mL was prepared in VNSS medium from the overnight culture. The PU-based surfaces were first placed in the plate wells, 3 mL of culture was added and then the microplates were incubated for biofilm development. At least three glass substrates of each surface were removed every 7 days for biofilm analysis. The growth medium was changed twice a week during the incubation period. Two independent biofilm formation assays, with at least three technical replicates each, were performed.

The removed glass substrates were removed and washed with a saline solution to eradicate unattached bacteria, and total cell counting was determined using a light microscope as indicated in Section 3.2. The biofilm architecture was also evaluated through a CLSM (Leica TCS SP5 II confocal laser scanning microscope, Leica Microsystems, Wetzlar, Germany) after 49 days. Biofilm samples were counterstained with Syto^®^ 9 (Thermo Fisher Scientific, Waltham, MA, USA) and then observed at an excitation wavelength of 488 nm (argon laser) using an HCX PL APO CS 40×/1.10 CORR water objective lens. Stacks of horizontal plane images (512 × 512 pixels, corresponding to 387.5 × 387.5 µm) with a z-step of 1 µm were acquired for each sample from at least five randomly chosen areas. For image analysis, the “Easy 3D” function of the IMARIS 9.1 software package (Bitplane, Zurich, Switzerland) was used for modelling in three dimensions (Figure 4). Three-dimensional rendering of the biofilms was also created from the same confocal stacks using the “Surpass” function of the IMARIS software (Figure S1 of Supplementary Material). The quantitative structural parameters of the biofilms—biovolume (µm^3^/µm^2^) and thickness (µm)—were calculated with the plug-in COMSTAT2 in ImageJ 1.48v software (U. S. National Institutes of Health, Bethesda, MD, USA).

### 3.5. In Vitro Transcriptional Activation of HsPPARγ, DrPPARγ and DrPXR

COS-1 cells were maintained in Dulbecco’s Modified Eagle’s Medium (DMEM, PAN-Biotech, Aidenbach, Bayern, Germany) with phenol red, and supplemented with 10% fetal bovine serum (PAN-Biotech, Aidenbach, Bayern, Germany) and 1% penicillin/streptomycin (PAN-Biotech, Aidenbach, Bayern, Germany) at 37 °C in a humidified chamber containing 5% CO_2_. Before transfection, cells were seeded in 24-well plates at a density of 2 × 10^5^ cells/mL. After 24 h, cells were transfected using 2 μL Lipofectamine 2000 (Invitrogen, Thermo Fisher Scientific, Waltham, MA, USA) and 0.5 μg of each construct, in Opti-MEM reduced serum medium (Gibco, Thermo Fisher Scientific, Waltham, MA, USA) to a final volume of 350 μL, as follows: (1) pBIND DrPPARγ LBD-Gal4 and pGL4.35 luciferase reporter vector; (2) pBIND HsPPARγ LBD-Gal4, and pGL4.35; and (3) pBIND DrPXR LBD-Gal4 and pGL4.35. Transfection procedures were carried out according to the manufacturer’s instructions. After 5 h of incubation, cells were washed with phosphate buffered saline and exposed to test compounds in a DMEM medium without phenol red and supplemented with 10% charcoal-stripped fetal bovine serum (PAN-Biotech, Aidenbach, Bayern, Germany) and 1% penicillin/streptomycin (PAN-Biotech, Aidenbach, Bayern, Germany). DMSO was used as a solvent to prepare test compound solutions and the final solvent concentration per well did not exceed 0.1%. Each test condition was assayed in duplicate. On the following day, cells were washed and gently lysed for 15 min at 37 °C and 90 rpm, using 100 μL of Passive Lysis Buffer (Promega, Madison, WI, USA). Firefly luciferase (pGL4.35) and Renilla luciferase (pBIND) luminescent activities were assessed using the Dual luciferase assay system according to manufacturer’s instructions (Promega) and measured with a Synergy HT Multi-Mode Microplate Reader (Biotek, Agilent Technologies, Santa Clara, CA, USA). Renilla luciferase, co-expressed with the LBD-Gal4 hybrid proteins, served as an internal control for transfection efficiency. Experiments were repeated three times.

### 3.6. Data Analysis

Transactivation assay data were obtained through the ratio of Firefly luciferase and Renilla luciferase activity and then normalized by dividing the ratio by the average of the solvent control (DMSO). Statistical analyses and graphs were performed in SigmaPlot 11. Student–Newman–Keuls ANOVA post hoc test (after one-way ANOVA) was used to compare groups. Significance was set at *p*-value (*p*) ≤ 0.05.

## 4. Conclusions

GBA26, a synthetic gallic acid derivative that was recently designed following a lead optimization strategy, exhibited highly effective AF activity against the settlement of *Mytillus galloprovincialis* adhesive larvae, both in solution and after incorporation in a polyurethane (PU) marine coating, with higher potency than the previous analog and the commercial biocide tralopyril [12]. GBA26 also had a higher LC_50_/EC_50_ ratio than the previous analog and tralopyril [12], thereby highlighting its safer profile against this hard fouler species.

As bioactivity against a single hard fouler may not be seen in assays against soft fouling, the impact of GBA26 on biofilm prevention and/or reduction was studied in this work. It was shown that GBA26 was able to prevent the formation of biofilms of *Pseudoalteromonas tunicata*, and also to promote the reduction in preformed biofilm of this representative marine bacterium. Under a hydrodynamic assay that simulates the marine environment, formulations of a marine PU-based coating containing varying contents of this AF agent showed promising in vitro anti-biofilm performances when tested against biofilms of the *P. tunicata* microfouler. The new PU-based marine coating containing 2 wt.% GBA26 and the trimethylolpropane triaziridine propionate (TZA) crosslinker provided the best long-term performance. The improved performance of this coating formulation compared to the one containing only a similar amount of GBA26 indicated that the compatibility of the compound in this polymer matrix, and the service life of the generated matrix, were improved due to the crosslinks with TZA.

Even though GBA26 was previously shown to have a safer profile than the previous analog and tralopyril on different trophic levels of aquatic organisms (mussel larvae, marine shrimp, and marine diatom [12]), possible interference of GBA26 on endocrine processes of aquatic organisms was assessed in this work to pursue a systematic ecotoxicological assessment [2]. All biocidal products require authorization before they can be placed on the market and the active substances contained in that biocidal product must be previously approved. In principle, active substances that fulfill the exclusion criteria will not be approved, which is the case with endocrine disruptors. It was observed in this study that this compound does not modulate the transcription of the selected NRs, at least in vitro and at low concentrations (≤10 µM). Although the present results support a lack of interaction of GBA26 with the nuclear receptors tested, caution should be taken and additional ecotoxicological assays should be performed in the future. These include a larger portfolio of NRs and tested taxonomic groups, together with chronic exposures combined with “omics” analysis.

Given the AF activity of GBA26-based PU marine coatings against the selected hard and soft foulers and the ecotoxicological studies performed, the next step will be to up-scale the synthesis of this simple compound in order to test the improved GBA26-coating formulation developed in this work in a real marine environment, so as to evaluate the formulation’s effectiveness on the whole biofouling community. Selecting simple, nature-inspired chemical structures during the first stages of the AF discovery process will be rewarding at this stage as large quantities will be possible to obtain and at a low cost [14]. The synthesis must also be adapted to obey the Twelve Principles of Green Chemistry, namely the use of eco-friendly solvents, replacement of hazardous reagents, and waste minimization. Furthermore, the starting material for the synthesis of this gallic acid derivative is a natural compound that may be obtained from grape waste extract, allowing for the valorization of winery industries waste. By extracting the starting material from grape waste and transforming it into value-added AF product, both tasks that apply green chemistry methodologies, this new AF compound may pave the way for new affordable and sustainable AF agents.

## Figures and Tables

**Table 1:** PU-based marine coating formulations containing GBA26.

Coating Formulation	Base/Curing Agent Ratio (wt.%)	GBA26 Content (wt.%)	CL ^1^ Content (wt.%)
GBA26-PU ^2^	9/1	1.05 ± 0.01	-
GBA26-PU		1.98 ± 0.01	-
GBA26/PU/CL		1.88 ± 0.01	1.08 ± 0.48

^1^ CL: trimethylolpropane triaziridine propionate crosslinker (TZA); ^2^ PU: Polyurethane.

## Supplementary Materials

The following supporting information can be downloaded at: https://www.mdpi.com/article/10.3390/md20080507/s1, Figure S1: Isosurface rendered 3D visualizations of *Pseudoalteromonas tunicata* biofilms formed on a surface protected with 1 wt.% GBA26/PU coating, a surface treated with 2 wt.% GBA26/PU coating, and a surface treated with 2 wt.% GBA26/PU/CL coating after 49 days. These images were obtained from the same confocal stacks used to create Figure 3 using the “Surpass” function of IMARIS software. Each square of the 3D grid corresponds to 20 × 20 μm.
