# Supplementary material for: Antifouling Marine Coatings with a Potentially Safer and Sustainable Synthetic Polyphenolic Derivative
Source: Mar Drugs. 2022 Aug 5;20(8):507. doi: 10.3390/md20080507 (PMC9409691; doi:10.3390/md20080507)
Supplement: Supplementary file 1 [file marinedrugs-20-00507-s001.zip › marinedrugs-1798073-supplementary.pdf]

Supplementary Material

Antifouling Marine Coatings with a Potentially Safer and Sustainable Synthetic Polyphenolic Derivative

Ana R. Neves[1,2], Luciana C. Gomes[3,4], Sara I. Faria[3,4], João Sousa[2], Raquel Ruivo[2], Inês Páscoa[2], Madalena Pinto[1,2], Emília Sousa[1,2], Miguel M. Santos[2,5], Elisabete R. Silva[6,7,*], Marta Correia-da-Silva[1,2,*], Filipe Mergulhão[3,4]

[1] Laboratory of Organic and Pharmaceutical Chemistry, Faculty of Pharmacy, University of Porto, Rua Jorge de Viterbo Ferreira 228, 4050-313 Porto, Portugal.

[2] Interdisciplinary Centre of Marine and Environmental Research (CIIMAR), University of Porto, Novo Edifício do Terminal de Cruzeiros do Porto de Leixões, Av. General Norton de Matos, s/n, 4450-208 Matosinhos, Portugal

[3] LEPABE—Laboratory for Process Engineering, Environment, Biotechnology and Energy, Faculty of Engineering, University of Porto, Rua Dr. Roberto Frias, 4200-465 Porto, Portugal.

[4] ALiCE - Associate Laboratory in Chemical Engineering, Faculty of Engineering, University of Porto, Rua Dr. Roberto Frias, 4200-465 Porto, Portugal.

[5] FCUP—Faculty of Sciences, University of Porto, Department of Biology, Porto, Portugal.

[6] BioISI—Biosystems & Integrative Sciences Institute, Faculty of Sciences, University of Lisboa, Campo Grande, 1749-016 Lisboa, Portugal.

[7] Departamento de Química e Bioquímica, Faculdade de Ciências, Universidade de Lisboa, Campo Grande, 1749-016 Lisboa, Portugal.

* Correspondence: ersilva@fc.ul.pt (coatings); m_correiadasilva@ff.up.pt (polyphenolic derivative)

1 wt.% GBA26/PU | **2 wt.% GBA26/PU** | **2 wt.% GBA26/PU/CL**

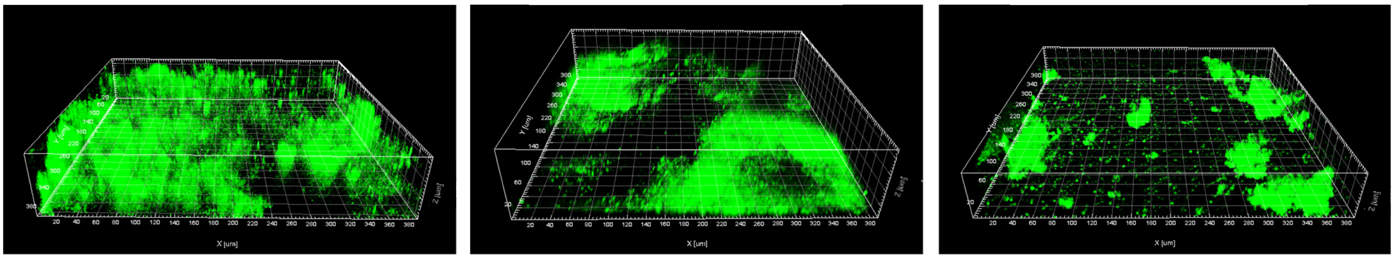

Figure S1. Isosurface rendered 3D visualizations of *Pseudoalteromonas tunicata* biofilms formed on a surface protected with 1 wt.% GBA26/PU coating, a surface treated with 2 wt.% GBA26/PU coating, and a surface treated with 2 wt.% GBA26/PU/CL coating after 49 days. These images were obtained from the same confocal stacks used to create Figure 3 using the “Surpass” function of IMARIS software. Each square of the 3D grid corresponds to 20 × 20 μm.
